# Quantifying how diagnostic test accuracy depends on threshold in a meta‐analysis

**DOI:** 10.1002/sim.8301

**Published:** 2019-09-30

**Authors:** Hayley E. Jones, Constantine A. Gatsonsis, Thomas A. Trikalinos, Nicky J. Welton, A.E. Ades

**Affiliations:** ^1^ Population Health Sciences, Bristol Medical School University of Bristol Bristol UK; ^2^ Department of Biostatistics, Center for Statistical Sciences Brown University School of Public Health Providence Rhode Island; ^3^ Center for Evidence Synthesis in Health Brown University School of Public Health Providence Rhode Island

**Keywords:** Box‐Cox transformation, evidence synthesis, ROC curve, sensitivity, specificity, test cutoff

## Abstract

Tests for disease often produce a continuous measure, such as the concentration of some biomarker in a blood sample. In clinical practice, a threshold *C* is selected such that results, say, greater than *C* are declared positive and those less than *C* negative. Measures of test accuracy such as sensitivity and specificity depend crucially on *C*, and the optimal value of this threshold is usually a key question for clinical practice. Standard methods for meta‐analysis of test accuracy (i) do not provide summary estimates of accuracy at each threshold, precluding selection of the optimal threshold, and furthermore, (ii) do not make use of all available data. We describe a multinomial meta‐analysis model that can take any number of pairs of sensitivity and specificity from each study and explicitly quantifies how accuracy depends on *C*. Our model assumes that some prespecified or Box‐Cox transformation of test results in the diseased and disease‐free populations has a logistic distribution. The Box‐Cox transformation parameter can be estimated from the data, allowing for a flexible range of underlying distributions. We parameterise in terms of the means and scale parameters of the two logistic distributions. In addition to credible intervals for the pooled sensitivity and specificity across all thresholds, we produce prediction intervals, allowing for between‐study heterogeneity in all parameters. We demonstrate the model using two case study meta‐analyses, examining the accuracy of tests for acute heart failure and preeclampsia. We show how the model can be extended to explore reasons for heterogeneity using study‐level covariates.

## 1 INTRODUCTION

Many diagnostic tests produce an explicit continuous measure, which is dichotomised at some threshold to call the result positive or negative. Identifying the optimal threshold to be used in practice is usually of key clinical importance. In addressing this question, standard methods for meta‐analysis of test accuracy^1^, ^2^, ^3^ have two major shortcomings. Firstly, these methods produce only a “summary” estimate of sensitivity and specificity and/or a summary receiver operating characteristic (ROC) curve: they do not explicitly quantify test accuracy at each possible threshold. Secondly, they synthesise only a single estimate of sensitivity and specificity from each study, despite studies very often reporting estimates at multiple thresholds.^4^ The presence of these additional data is widely regarded as problematic, due to the additional complexities in data synthesis. However, within‐study information on how test accuracy varies with threshold could clearly be extremely valuable, both for quantifying the average sensitivity and specificity across all thresholds and for disentangling heterogeneity due to varying thresholds from that due to other factors.

A simple approach to addressing both problems is to perform a separate meta‐analysis of the data at each threshold or for groups of similar thresholds. The Cochrane Handbook for Systematic Reviews of Diagnostic Test Accuracy notes that “Each study can contribute to one or more analyses depending on what thresholds it reports”.^5^ This will produce summary estimates of the sensitivity and specificity of the test at each threshold in the data set. However, case studies have demonstrated bias in these estimates if some studies only report accuracy measures at data‐driven “optimal” thresholds.^6^, ^7^, ^8^ Confidence or credible intervals will also be very wide for thresholds with limited data. It might be possible to address these problems through imputation of missing data in each study prior to meta‐analysis,^7^, ^8^ although accounting for uncertainty in these imputations requires extra steps.^8^ An additional problem also remains: if higher values increase the likelihood of a disease, by definition, sensitivity must reduce and specificity increase with increasing threshold. However, these relationships will not necessarily hold in the summary estimates.

The alternative is a single unified analysis of all available data. This will enable “borrowing of strength” across thresholds and produce estimates that conform to the known relationship between threshold, sensitivity, and specificity. Various models have been proposed for such a unified analysis. Some of these produce a summary ROC curve but not estimates of sensitivity and specificity relating to specific thresholds.^9^, ^10^, ^11^ Others were devised for synthesis of ordinal test results with a small number of categories.^12^, ^13^, ^14^ Extensions of these models for truly continuous test results would require very large numbers of parameters to be estimated. For example, Riley et al proposed a 2*K dimensional multivariate normal model, where *K* is the total number of distinct thresholds included in the meta‐analysis, but noted that this will often not be estimable.^15^

An alternative approach is to explicitly model sensitivity and specificity as functions of threshold.^4^, ^15^ However, there is a lack of clarity on which function of threshold is the most appropriate. Steinhauser et al^4^ and Hoyer et al^16^ proposed unified models that assume that test results in the diseased and disease‐free populations have a prespecified distributional form, for example, log‐normal. A criticism is that the appropriate choice of distribution might not be known in advance, however.^8^ Hoyer et al note that it would be a “definite advantage” if the distributions could be “estimated simultaneously together with the other model parameters”.^16^

We present a new model that, compared with previous approaches, has most in common with but potential advantages over that suggested by Steinhauser et al.^4^ We model the exact multinomial likelihoods of the spread of test results across categories defined by thresholds, rather than requiring the normal approximations used by Steinhauser et al.^4^ This approach automatically accounts for within‐study correlations resulting from studies reporting at more than one threshold and should perform better with small counts.^2^ We also relax the assumption that the appropriate distributional form is known, assuming only that some Box‐Cox transformation of test results in the two populations has a logistic distribution. The Box‐Cox transformation parameter can be estimated from the data. Our model is parameterised directly in terms of the means and scale parameters of these two logistic distributions, and is easily extended to allow for study‐level covariates impacting upon any of these four parameters.

We first describe the model in Section 2, including the extended version to include study‐level covariates. In Section 3, we describe two case study data sets, to which we then apply the model in Section 4, before concluding in Section 5.

## 2 A GENERAL FLEXIBLE MODEL STRUCTURE

We describe a flexible model that is straightforward to fit in a Bayesian framework using Markov chain Monte Carlo (MCMC) simulation software, such as WinBUGS^17^ or OpenBUGS.

### 2.1 Notation and within‐study model

We consider the case where each study, *i*, reports estimates of sensitivity and specificity at *T*_*i*_ distinct thresholds; or, equivalently, directly reports count data in a form such as Table 1. *T*_*i*_ might be equal to one in some studies, ie, it is not required that all studies contribute more than one pair of data points. We will assume throughout that the true disease state is known for all individuals, through application of some gold standard test. We denote the total number of individuals without and with the disease in study *i* by *N*_*i*1_ and *N*_*i*2_, respectively.

**Table 1:** Test accuracy data from a study, indexed i, providing estimates of sensitivity and specificity at *T*_*i*_ distinct thresholds (*C*_*i*1_,…, $C_{iT_{i}}$)

Population	Total number of patients	Number with test result > *C*_*i*1_	⋯	Number with test result > $C_{iT_{i}}$
Disease‐free	*N*_*i*1_	*x*_*i*11_	⋯	$x_{i1T_{i}}$
Diseased	*N*_*i*2_	*x*_*i*21_	⋯	$x_{i2T_{i}}$

We assume that higher values of the continuous test result are associated with increased likelihood of disease, such that a “positive” test result is one that falls above a given threshold. At each threshold *C*_*it*_, *t* = 1,…,*T*_*i*_, we denote the number of false positive and true positive individuals by *x*_*i*1*t*_ and *x*_*i*2*t*_, respectively (Table 1). These counts must, by definition, be monotonically decreasing with *t*, a property which the model should reflect.

In each study, we can subdivide each patient population (*N*_*i*1_ and *N*_*i*2_) into (*T*_*i*_ + 1) mutually exclusive groups, with test results falling below *C*_*i*1_, between *C*_*it*_ and *C*_*i,t* + 1_ (*t* = 1,…,T_*i*_‐1), and above $C_{i,T_{i}}$. The distribution of each of the two sets of results across these groups is multinomial. Conditional on the underlying probability parameters, the two multinomial distributions for each study *i* are independent of each other.

For model fitting purposes, it is convenient to use the binomial factorisation of these multinomial distributions,^18^ in which they are written as a series of conditionally independent binomial distributions, ie, for population *j* = 1 (disease‐free) and *j* = 2 (diseased):

$$\begin{array}{ll} x_{\text{ij}1} & ∼\text{Binomial}\left(N_{\text{ij}},{\text{pr}}_{\text{ij}1}\right)\\ x_{\text{ijt}}|x_{\text{ij},t-1} & ∼\text{Binomial}\left(x_{\text{ij},t-1},\frac{{\text{pr}}_{\text{ijt}}}{{\text{pr}}_{\text{ij},t-1}}\right),\;t=2,\ldots ,T_{i}, \end{array}$$

where *pr*_*i*1*t*_ and *pr*_*i*2*t*_ are the false positive rate ( *fpr*) (= 1‐ specificity) and true positive rate (*tpr*) (= sensitivity) at threshold *C*_*it*_ in study *i*. By definition, *pr*_*i*1*t*_ and *pr*_*i*2*t*_ monotonically decrease with increasing *t* and lie in [0,1], such that each Binomial probability parameter is unconstrained within the interval [0,1]. This parameterisation obviates the need to reexpress the Table 1 data as numbers of patients falling between each threshold value, and allows the same model code to be applied to studies with binomial (*T*_*i*_ *=* 1) and multinomial (*T*_*i*_ *>* 1) likelihoods.

We wish to specify the *tpr*s and *fpr*s as functions of threshold. The appropriate function depends on the distribution of continuous test results in the disease‐free and diseased populations. We will assume that there exists some monotonic transformation, g(), that transforms test results in each of the two populations to either a normal or logistic distribution. This is the most common assumption made in the fitting of a smooth line to an empirical ROC curve.^19^ We will work on the basis of logistic distributions throughout, which are similar to normal and more computationally convenient (leading to logit rather than probit link functions). We assume for now that the same g() applies to both distributions and to all studies in the meta‐analysis, but will discuss relaxing this assumption later (Section 5).

Let us denote the continuous test results of disease‐free and diseased individuals in the *i*th study by *y*_*i*1*k*_ and *y*_*i*2*k*_ respectively, where *k* is an index for individual. We assume that

$$g\left(y_{\text{ijk}}\right)∼\text{Logistic}\left(\mu_{\text{ij}},\sigma_{\text{ij}}\right),\;j=1,2,$$

where *μ*_*ij*_ and *σ*_*ij*_ denote mean and scale parameters for disease status group *j*. As g() is monotonic, *pr*_ijt_ ≡ Pr(*y*_*ijk*_ ≥ *C*_*it*_) =  Pr (*g*(*y*_*ijk*_) ≥ *g*(*C*_*it*_)). It follows from the cumulative distribution function of the logistic distribution that the *fpr*s and *tpr*s at a threshold of *C*_*it*_ in study *i* are defined as follows:

(1) $$\text{logit}\left({\text{pr}}_{\text{ijt}}\right)=\frac{\mu_{\text{ij}}-g\left(C_{\text{it}}\right)}{\sigma_{\text{ij}}},\;t=1,\ldots ,T_{i}\;;j=1,2.$$

### 2.2 Choice of transformation function, g()

We see from Equation (1) that to explicitly model the dependence of *pr*_*ijt*_ on *C*_*it*_, we need to move beyond the “semiparametric” or “distribution‐free” approach often used to fit smooth ROC curves,^19^ in which the transformation function g() remains unspecified. A fully parametric model is required. In particular, specifying logit (*pr*_*i*1*t*_) and logit (*pr*_*i*2*t*_) as linear functions of untransformed *C*_*it*_ (eg, the work of Riley et al^15^) would implicitly assume that g() is the identity function, that is, that the test results in the diseased and disease‐free populations have symmetric, logistic distributions.

We might be comfortable to prespecify an appropriate transformation, g(). This could be informed by inspection of the distributions of test results from a laboratory or from one or more study publications. Often in practice, assuming g() is the natural logarithm (subsequently referred to simply as “log()”) will be a reasonable approximation for positive valued test results, which are often right skewed.^20^ This corresponds to assuming a log‐logistic distribution for each set of test results, one of the distributional forms considered by both Steinhauser et al^4^ and Hoyer et al.^16^

If, however, the analyst is not confident about the most appropriate transformation or would like to assess sensitivity of results to this assumption, we propose using a more flexible approach. We assume only that g() is one of the set of Box‐Cox transformations, defined by

$$g\left(C_{\text{it}}\right)=\left\{\begin{array}{c} \left(C_{\text{it}}^{\lambda }-1\right)/\lambda ,\;\text{if}\;\lambda \neq 0\;\text{and}\\ \;\text{log}\left(C_{\text{it}}\right),\;\text{if}\;\lambda =0. \end{array}\right.$$

This reduces to the assumption of logistic distributions of underlying test results when *λ* = 1. As *λ* decreases from 1, this indicates an increasing degree of right skew of the underlying distributions, with λ = 0 corresponding to log‐logistic distributions.

The transformation parameter *λ* can be estimated with uncertainty from the data. This approach was proposed by Zou and Hall^21^ for the estimation of ROC curves in a single study but to our knowledge has not been previously applied in a meta‐analysis setting.

### 2.3 Between‐study model

We assume that, across studies, *μ*_*ij*_ is normally distributed with mean *m*_*μj*_ and variance $\tau_{\mu j}^{2}$, for each population *j* = 1,2. Similarly, log(*σ*_*ij*_) is assumed to be normally distributed with mean *m*_*σj*_ and variance $\tau_{\sigma j}^{2}$.

We would generally anticipate some correlations across these four sets of random effects. Any between‐study correlation structure might be specified. Here, we describe three, each of which we will apply to our case study data sets in Section 4.

*(i) Full correlation matrix*

To allow for all possible between‐study correlations, we can fit a full quadrivariate normal distribution with six different correlation parameters:

(2) $$\left(\begin{array}{c} \mu_{i1}\\ \mu_{i2}\\ \log\left(\sigma_{i1}\right)\\ \log\left(\sigma_{i2}\right) \end{array}\right)∼\text{MVN}\left(\left(\begin{array}{c} m_{\mu 1}\\ m_{\mu 2}\\ m_{\sigma 1}\\ m_{\sigma 2} \end{array}\right),\left(\begin{array}{cccc} \tau_{\mu 1}^{2} & \rho_{\mu }\tau_{\mu 1}\tau_{\mu 2}\; & \rho_{\mu 1\sigma 1}\tau_{\mu 1}\tau_{\sigma 1}\; & \rho_{\mu 1\sigma 2}\tau_{\mu 1}\tau_{\sigma 2}\\ \rho_{\mu }\tau_{\mu 1}\tau_{\mu 2}\; & \tau_{\mu 2}^{2}\; & \rho_{\mu 2\sigma 1}\tau_{\mu 2}\tau_{\sigma 1}\; & \rho_{\mu 2\sigma 2}\tau_{\mu 2}\tau_{\sigma 2}\\ \rho_{\mu 1\sigma 1}\tau_{\mu 1}\tau_{\sigma 1}\; & \rho_{\mu 2\sigma 1}\tau_{\mu 2}\tau_{\sigma 1}\; & \tau_{\sigma 1}^{2}\; & \rho_{\sigma }\tau_{\sigma 1}\tau_{\sigma 2}\\ \rho_{\mu 1\sigma 2}\tau_{\mu 1}\tau_{\sigma 2}\; & \rho_{\mu 2\sigma 2}\tau_{\mu 2}\tau_{\sigma 2}\; & \rho_{\sigma }\tau_{\sigma 1}\tau_{\sigma 2}\; & \tau_{\sigma 2}^{2} \end{array}\right)\right).$$

In WinBUGS, this can be fitted using a product normal formulation, which we describe in Appendix.

*(ii) Structured correlation matrix*

As correlation parameters in multivariate meta‐analysis models can be difficult to estimate, it is desirable to reduce the number of these to be estimated by prespecifying a realistic correlation structure.

One simplifying set of assumptions might be that all correlations arise through dependencies between the following three pairs of parameters: *μ*_*i*1_ and *μ*_*i*2_; *μ*_*i*1_ and log(*σ*_*i*1_); *μ*_*i*2_ and log(*σ*_*i*2_). In general, we might expect *μ*_*i*1_ and *μ*_*i*2_ to be positively correlated across studies. For example, study‐level factors might raise or lower the expected test result in both the diseased and disease‐free populations. We will denote this correlation by *ρ*_*μ*_ (as in (2)). Study‐specific log‐scale parameters might also be expected to be positively correlated with means in the same patient group. We will assume that this correlation is the same in the diseased and disease‐free populations and will denote it by *ρ*_*μσ*_. We hypothesise that any other correlations between random effects (for example, between *μ*_*i*1_ and log(*σ*_*i*2_)) are likely to be induced through *ρ*_*μ*_ and *ρ*_*μσ*_.

The corresponding quadrivariate normal distribution can be written as four conditionally independent univariate distributions as follows:

$$\begin{array}{ll} \mu_{i1} & ∼\text{Normal}\left(m_{\mu 1},\tau_{\mu 1}^{2}\right)\\ \mu_{i2}|\mu_{i1} & ∼\text{Normal}\left(m_{\mu 2}+\rho_{\mu }\frac{\tau_{\mu 2}}{\tau_{\mu 1}}\left(\mu_{i1}-m_{\mu 1}\right),\left(1-\rho_{\mu }^{2}\right)\tau_{\mu 2}^{2}\right)\\ \log\left(\sigma_{\text{ij}}\right)|\mu_{\text{ij}} & ∼\text{Normal}\left(m_{\sigma j}+\rho_{\mu \sigma }\frac{\tau_{\sigma j}}{\tau_{\mu j}}\left(\mu_{\text{ij}}-m_{\mu j}\right),\left(1-\rho_{\mu \sigma }^{2}\right)\tau_{\sigma j}^{2}\right),\;j=1,2. \end{array}$$

*(iii) Independence*

For completeness, we include a model with four independent sets of random effects, ie, with all six correlation parameters in (2) equal to 0:

$$\begin{array}{ll} \mu_{\text{ij}} & ∼N\left(m_{\mu j},\tau_{\mu j}^{2}\right),\;\text{and}\\ \log\left(\sigma_{\text{ij}}\right) & ∼N\left(m_{\sigma j},\tau_{\sigma j}^{2}\right)\;\text{for}\;j=1,2. \end{array}$$

### 2.4 Inclusion of study‐level covariates

As in any meta‐analysis, potential reasons for heterogeneity across studies in diagnostic test accuracy should be explored where possible, rather than simply accommodated using random effects. It is straightforward to extend our model to include study‐level covariates, acting on either the location *μ*_*ij*_ and/or log‐scale log(*σ*_*ij*_) parameters. Furthermore, while it can be difficult to hypothesise how sensitivity and specificity might vary according to study characteristics, it seems natural to consider how the “average” test result or the spread of test results in either population might be affected.

A generalised version of the “full” model (Equation (2)) is as follows, where **z**_**ir**_ are vectors of study‐level covariates and **α**_**r**_ are vectors of meta‐regression coefficients to be estimated, *r* = 1,…,4:

(3) $$\left(\begin{array}{c} \mu_{i1}\\ \mu_{i2}\\ \log\left(\sigma_{i1}\right)\\ \log\left(\sigma_{i2}\right) \end{array}\right)∼\text{MVN}\left(\left(\begin{array}{c} m_{\mu 1}+\alpha_{1}{\;}^{\prime }\;z_{i1}\\ m_{\mu 2}+\alpha_{2}{\;}^{\prime }\;z_{i2}\\ m_{\sigma 1}+\alpha_{3}{\;}^{\prime }\;z_{i3}\\ m_{\sigma 2}+\alpha_{4}{\;}^{\prime }\;z_{i4} \end{array}\right),\left(\begin{array}{cccc} \tau_{\mu 1}^{2}\; & \rho_{\mu }\tau_{\mu 1}\tau_{\mu 2}\; & \rho_{\mu 1\sigma 1}\tau_{\mu 1}\tau_{\sigma 1}\; & \rho_{\mu 1\sigma 2}\tau_{\mu 1}\tau_{\sigma 2}\\ \rho_{\mu }\tau_{\mu 1}\tau_{\mu 2}\; & \tau_{\mu 2}^{2}\; & \rho_{\mu 2\sigma 1}\tau_{\mu 2}\tau_{\sigma 1}\; & \rho_{\mu 2\sigma 2}\tau_{\mu 2}\tau_{\sigma 2}\\ \rho_{\mu 1\sigma 1}\tau_{\mu 1}\tau_{\sigma 1}\; & \rho_{\mu 2\sigma 1}\tau_{\mu 2}\tau_{\sigma 1}\; & \tau_{\sigma 1}^{2}\; & \rho_{\sigma }\tau_{\sigma 1}\tau_{\sigma 2}\\ \rho_{\mu 1\sigma 2}\tau_{\mu 1}\tau_{\sigma 2}\; & \rho_{\mu 2\sigma 2}\tau_{\mu 2}\tau_{\sigma 2}\; & \rho_{\sigma }\tau_{\sigma 1}\tau_{\sigma 2}\; & \tau_{\sigma 2}^{2} \end{array}\right)\right).$$

Special cases include **z**_**ir**_ = **z**_**i**_, *r* = 1,…,4, whereby the same set of study‐level covariates is hypothesised to be associated with all four sets of random effects, and **z**_**ir**_ = 0 for some *r,* whereby we hypothesise associations of covariates with only a subset of the random effects.

## 3 CASE STUDY DATA SETS

We now describe two case study data sets, before fitting our model to each of these in Section 4.

***Example 1:* Brain natriuretic peptide (BNP) for diagnosis of acute heart failure**

Roberts et al^22^ performed a systematic review of the accuracy of brain natriuretic peptide (BNP) in diagnosing acute heart failure in adults presenting in an acute care setting with dyspnoea. The authors extracted measures of the accuracy of BNP, relative to a reference standard of retrospective review or final hospital diagnosis, from 26 studies of consecutive or randomly selected patients.^22^ Many of these studies reported sensitivity and specificity at more than one threshold.

By checking each of the original study publications, we found that additional data were often available. In some studies, these were not displayed in tables but were, however, shown on ROC plots, on which the thresholds corresponding to particular points on the curve had been marked. We extracted sensitivity and specificity estimates from these plots using the DigitizeIt software (http://www.digitizeit.de/).

**Figure 1:**
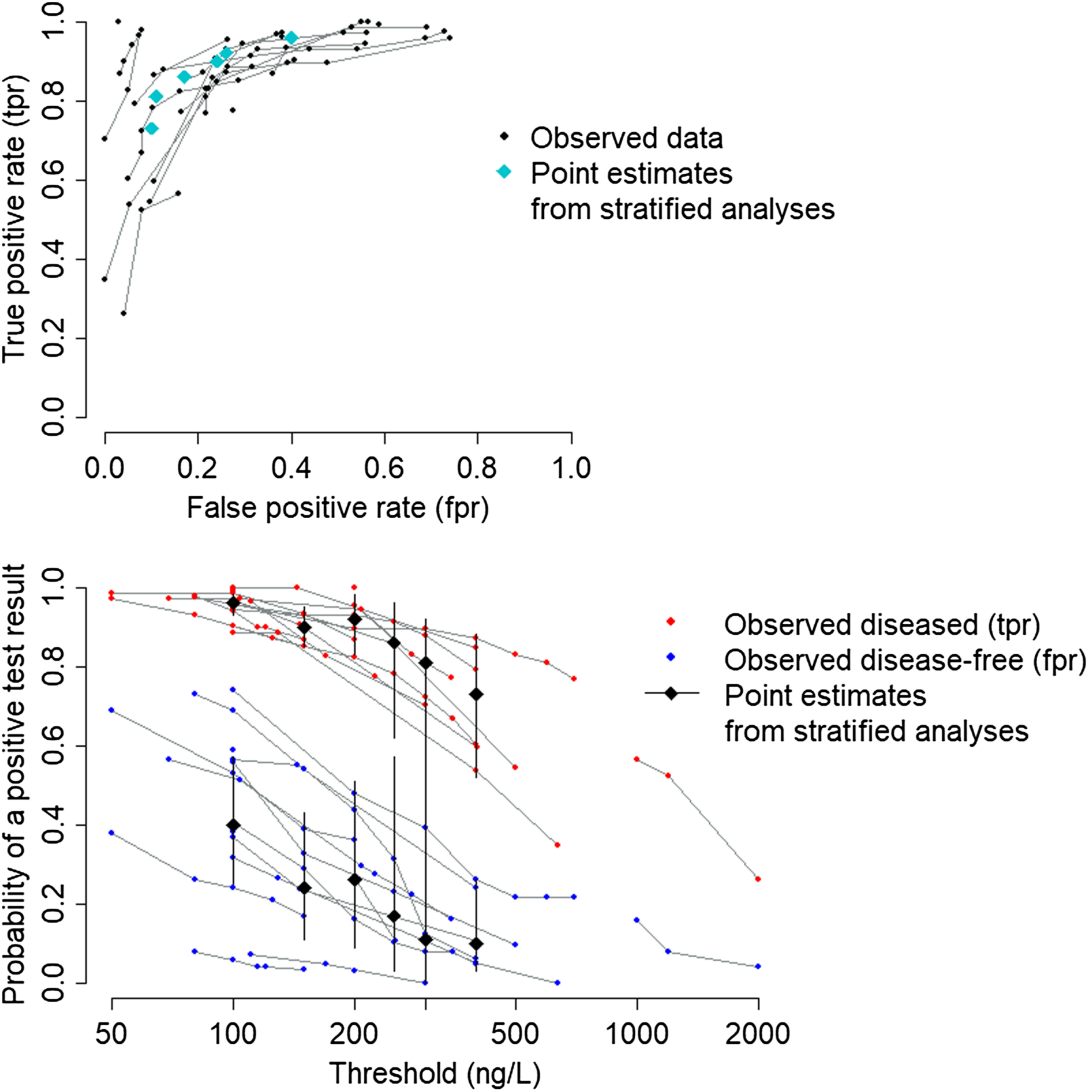
Observed data on the accuracy of Brain Natriuretic Peptide (Triage assay only) in diagnosing acute heart failure across the full observed range of thresholds. Points from the same study are joined. tpr = true positive rate (sensitivity), fpr = false positive rate (1‐specificity). Also shown are point estimates with 95% credible intervals from a series of stratified bivariate meta‐analyses, in which similar thresholds are grouped and analysed together [Colour figure can be viewed at http://wileyonlinelibrary.com]

It is recognised that a given BNP measurement on one assay might not translate directly to the same value on other assays.^23^ For this reason, we restrict our analyses to the 18 studies that assessed the accuracy of the Triage assay (Biosite Inc, San Diego). In total, the final data set consisted of 66 pairs of sensitivity and specificity from these 18 studies, ranging from a single pair (four studies) to seven (three studies). The data are displayed in two formats in Figure 1: on the ROC plane on the left, whereas on the right, we display how the probability of a positive test result for each of the two groups of patients depends on the threshold used. The data are available from https://wiley.figshare.com.

As noted above, a common approach to synthesising data with multiple thresholds, applied by authors including Roberts et al,^22^ is to group the data into categories with similar thresholds and perform a number of stratified analyses. To demonstrate this approach, we rounded all thresholds to the nearest 50 and performed stratified analyses: for each threshold with at least four contributing studies, we fitted the standard bivariate meta‐analysis model^1^, ^2^ in WinBUGS.^17^ Summary results are shown on Figure 1. We see that these stratified analyses produce estimates of the *tpr* and *fpr* that do not reduce monotonically with increasing threshold. This problem is masked in the ROC plot (Figure 1, top panel) but clearly visible when we plot summary estimates against explicit threshold values (bottom panel). Furthermore, credible intervals are very wide and it is not possible to estimate the accuracy of BNP across all threshold values.

There are several potential factors that might influence the accuracy of BNP as a test for acute heart failure. For example, Rogers et al^24^ found age, gender, ethnicity, body mass index, blood urine nitrogen, and creatinine to all be associated with BNP levels independently of heart failure. We extracted the average age of patients in each study to explore whether the accuracy of the test varied by this factor (values available in the https://wiley.figshare.com file).

***Example 2:* Spot protein to creatinine ratio (PCR) for diagnosis of preeclampsia**

**Figure 2:**
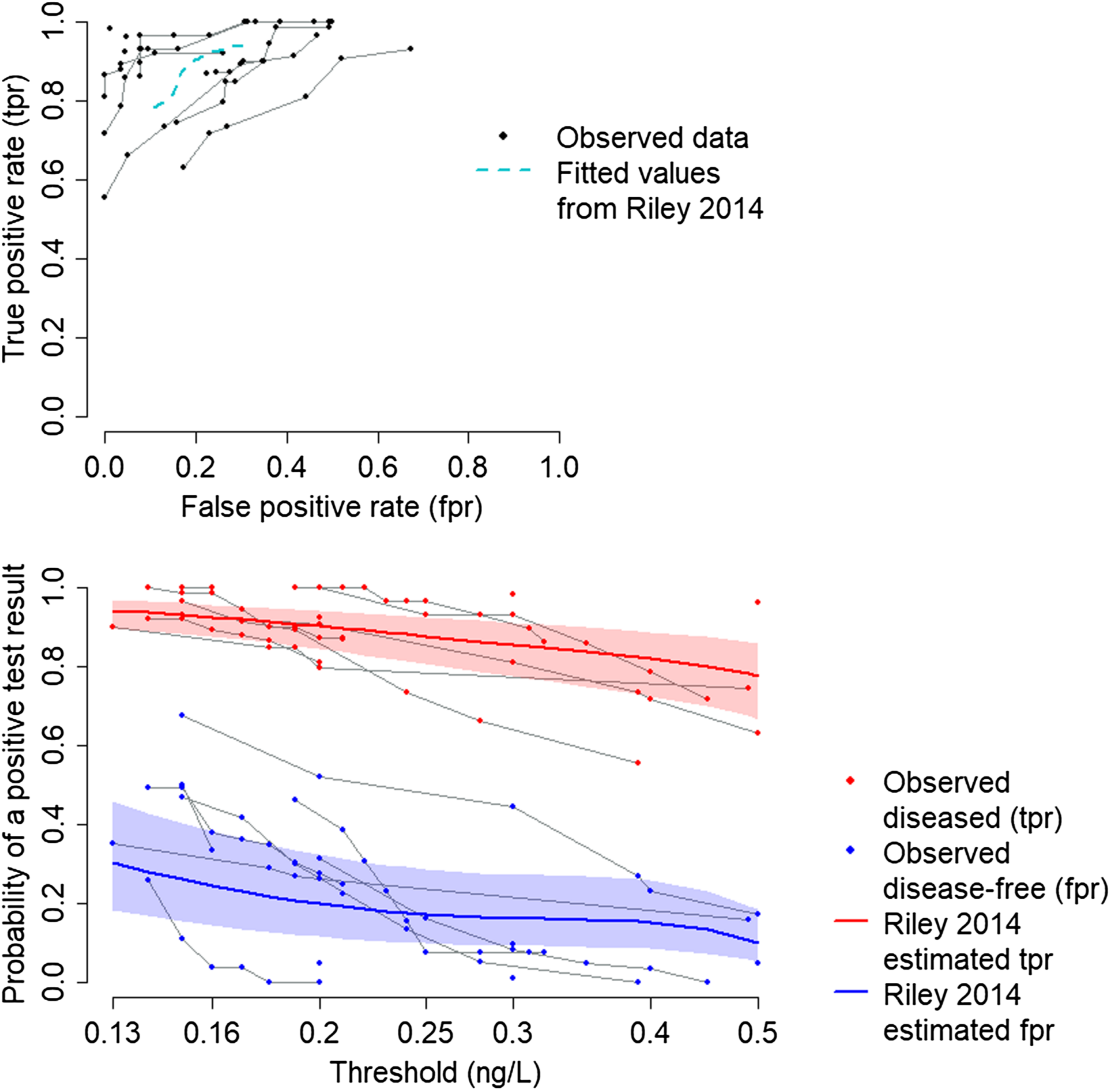
Observed data on the accuracy of spot urinary protein to creatinine ratio in detecting significant proteinuria in suspected preeclampsia. Points from the same study are joined. tpr = true positive rate (sensitivity), fpr = false positive rate (1‐specificity). Also shown are summary point estimates with 95% confidence intervals from an analysis by Riley et al^15^ [Colour figure can be viewed at http://wileyonlinelibrary.com]

Morris et al^25^ systematically reviewed the literature for studies assessing the diagnostic accuracy of spot urinary protein to creatinine ratio (PCR) in detecting significant proteinuria in pregnant women with suspected preeclampsia. Significant proteinuria is defined as ≥0.3 g/24 hours, the “gold standard” test for which is a 24‐hour urine collection. Data were extracted from 13 studies, each of which reported sensitivity and specificity estimates at between one (five studies) and nine (one study) thresholds.^25^ The data are displayed in Figure 2 and are available in full from Morris et al.^25^

Riley et al^15^ previously analysed this data set using a multivariate normal meta‐regression approach, in which logit (sensitivity) and logit (specificity) were modelled as polynomial functions of threshold, *C*. They found a cubic relationship with threshold to fit the data best. We show the summary estimates from their analysis on Figure 2 (point estimates and 95% CIs extracted from table 4 of Riley et al^15^). We see that the implied summary ROC curve is not concave and does not seem to fully capture the relationship between the *tpr*s and *fpr*s and threshold.

## 4 APPLICATION TO CASE STUDY DATA SETS

We now fit our proposed model to each of the two case study data sets using WinBUGS.^17^ We begin by fitting models with no study‐level covariates, then also explore whether heterogeneity can be explained by average patient age in the BNP data set. We gave Normal (0,10^2^) prior distributions to all means (*m*_*μj*_,*m*_*σj*_) and meta‐regression coefficients, Uniform (0,5) distributions to between‐study standard deviations (*τ*_*μj*_,*τ*_*σj*_), and Uniform(−1,1) prior distributions to any between‐study correlation parameters. We will compare results from models in which g() is prespecified with models in which the transformation function *λ* is estimated from the data (Section 2.2). For the latter, we assigned a Uniform(−3, 3) prior to *λ*. We also performed sensitivity analyses with a Uniform(1,10) prior distribution for λ, as used previously in analyses by O'Malley and Zou.^26^ WinBUGS code is available from https://wiley.figshare.com.

***Example 1:* B‐type natriuretic peptide for diagnosis of acute heart failure**

As many of the articles in this systematic review noted that BNP values are right skewed and used the natural logarithm of BNP values in their own analyses, eg, Karmpaliotis et al,^27^ Dokainish et al,^28^ and Davis et al,^29^ we first assumed g = log() and fitted the three between‐study models described in Section 2.3. Model fit penalising for complexity was compared using the Deviance Information Criterion (DIC).^30^ Models with lower values of the DIC are preferred.

As shown in Table 2, differences in DIC across the three correlation structures (Models 1‐3) were minimal. Notably, this is despite strong evidence of a positive correlation between the *μ*_*i*1_ and μ_*i*2_ (Model 2 estimate of *ρ*_*μ*_ = 0.80, 95% credible interval, Cr‐I, 0.24 to 0.99). In the absence of any reduction in DIC from modelling between‐study correlations, arguably the independence model is preferred for this data set.

**Table 2:** Comparison of model fit to the Brain natriuretic peptide data, according to the Deviance Information Criteria (DIC). $\bar{D}$= mean residual deviance, pD = effective number of parameters, DIC = $\bar{D}$+ pD

Model	Correlation structure	Transformation, g()	Study level covariates	$\bar{D}$	pD	DIC
1	Full correlation matrix	log()	None	216.7	41.0	257.7
2	Structured correlation matrix	log()	None	215.0	40.8	255.8
3	Independence	log()	None	214.1	42.5	256.6
4	Independence	Box‐Cox with unknown *λ*	None	211.3	43.6	254.9
5	Independence	log()	*μ*_*i*1_ and *μ*_*i*2_ regressed on average patient age	214.7	42.9	257.6

We then extended the independence model (Model 3) to estimate the best fitting Box‐Cox transformation parameter λ, rather than assuming g = log(). *λ* was estimated to be 0.23 (95% Cr‐I 0.10, 0.34), indicating that the underlying distributions of test results are slightly less right‐skewed than log‐logistic (λ = 0). This estimate was not sensitive to the choice of prior. As shown in Table 2 (Model 4), this model fitted the data marginally better as measured by the mean residual deviance, but with a minimal reduction in DIC (1.7 points).

Summary *fpr*s and *tpr*s for each model were calculated by evaluating Equation (1) at the means of the four sets of random effects, ie, for any threshold *C*:

$$\begin{array}{l} \text{logit}\left(\text{fpr}\left(C\right)\right)=\frac{m_{\mu 1}-g\left(C\right)}{\exp\left(m_{\sigma 1}\right)}\\ \text{logit}\left(\text{tpr}\left(C\right)\right)=\frac{m_{\mu 2}-g\left(C\right)}{\exp\left(m_{\sigma 2}\right)}. \end{array}$$

**Figure 3:**
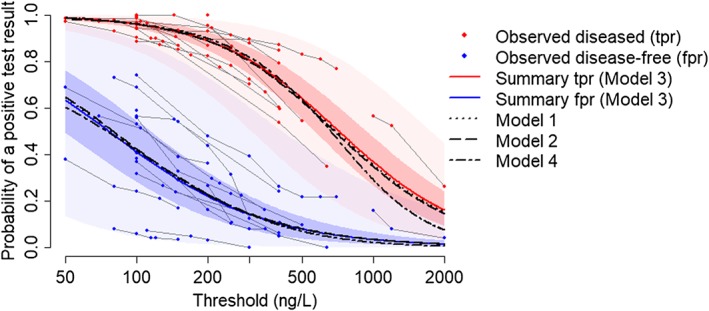
Summary true positive rate (tpr) and false positive rate (fpr) estimates (Models 1‐4) for the Brain natriuretic peptide data across the full range of thresholds. 95% credible intervals and prediction intervals shown are from Model 3 [Colour figure can be viewed at http://wileyonlinelibrary.com]

As shown on Figure 3, these were generally reassuringly similar across models, particularly across the range of thresholds encompassing most of the data: between thresholds of 100 and 500, the maximum absolute difference in summary *tpr* and *fpr* estimates across models was 1% and 2%, respectively. Model 4 provided substantially lower summary estimates of the *tpr* at very high thresholds (>5% absolute difference for thresholds above 780), where the data are very sparse. Compared with the stratified meta‐analyses of data at similar thresholds (Figure 1), the estimates of *tpr* and *fpr* are seen to be coherent (reducing as threshold increases) and more precise.

By drawing predictions for a “new” study population from each set of random effects, and calculating the *tpr* and *fpr* at these predicted values, we also generated 95% prediction intervals.^31^ For Model 3, Figure 3 shows prediction intervals in addition to Cr‐Is for summary estimates. The very wide prediction intervals, especially for the *fpr* at lower thresholds, illustrate that there is a large amount of between‐study heterogeneity that is not explained by variation in thresholds.

For comparison, we fitted the model proposed by Steinhauser et al^4^ to the same data set, using the “diagmeta” R package.^32^ A comparison of results with our “Model 3” is provided in the supplementary material. Summary *fpr* estimates with 95% CIs were very similar to estimates and Cr‐Is from our Model 3. Across thresholds, diagmeta estimated a slightly higher (up to 3%) summary *tpr* than our model. See Discussion for possible explanations.

**Figure 4:**
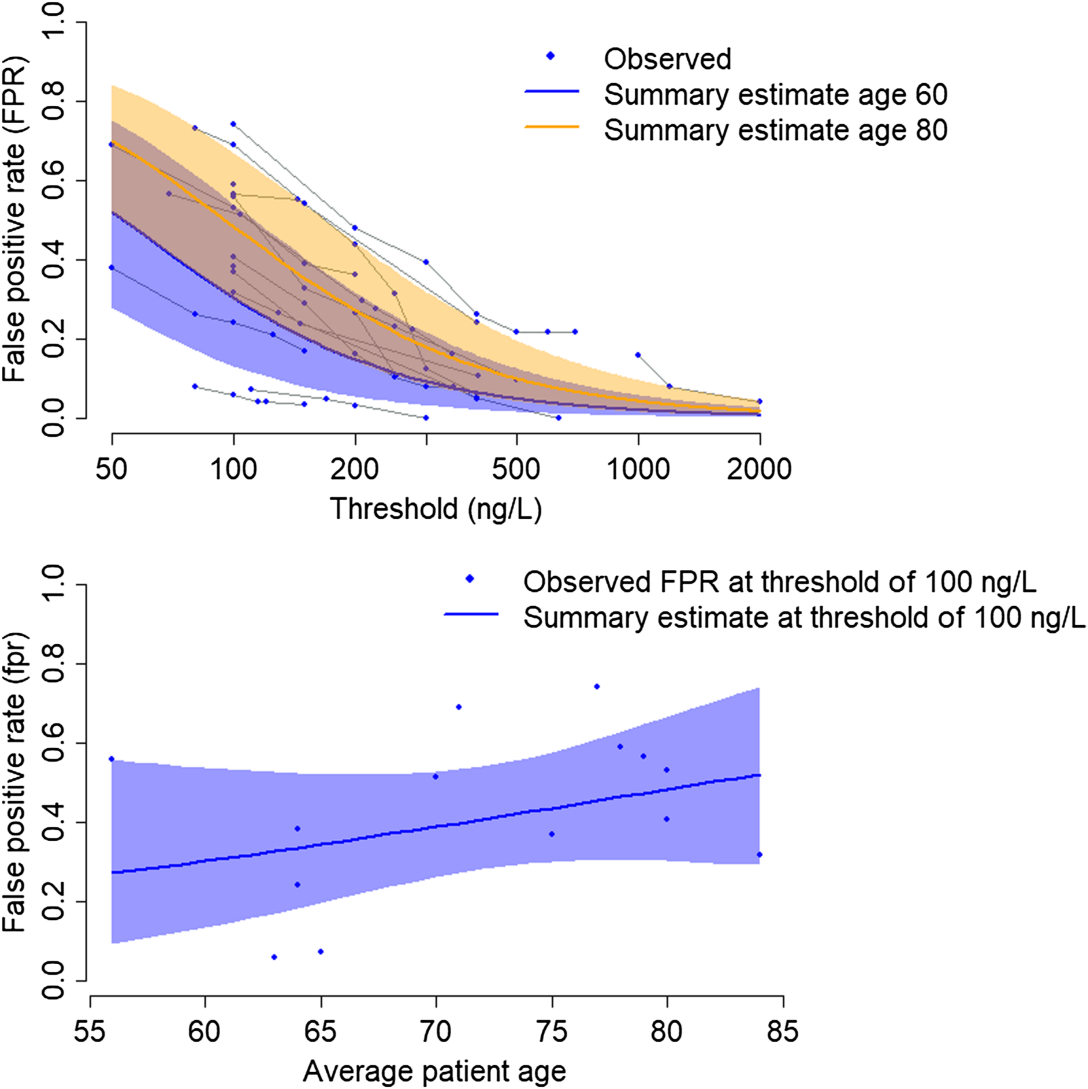
Relationship between average patient age and false positive rate of Brain Natriuretic Peptide (Triage assay) in diagnosing acute heart failure (Model 5 results). Top panel: summary false positive rate across all thresholds for age 60 and age 80. Bottom panel: summary false positive rate at a threshold of 100 ng/litre, by average patient age. Shaded areas are 95% credible intervals [Colour figure can be viewed at http://wileyonlinelibrary.com]

In an additional analysis (Model 5), we explored whether any of the between‐study heterogeneity could be explained by average patient age. Several studies have noted that BNP tends to increase with age.^33^, ^34^ We fitted an extended version of Model 3 to assess whether average patient age was associated with the study‐level location parameters, *μ*_*i*1_ and *μ*_*i*2_. Specifically, in Equation (3), we set **z**_**i1**_ = **z**_**i2**_ = (centered) average patient age and **z**_**i3**_ = **z**_**i4**_ = 0. As we built upon Model 3, all correlation parameters in Equation (3) were also set to zero. Among patients without acute heart failure, the model estimated that a 5‐year increase in average patient age was associated with a 15% increase in mean BNP, but the statistical evidence for this finding was weak (ratio = 1.15, 95% Cr‐I 0.90 to 1.51). As shown on Figure 4, this estimated dependence of *μ*_*i*1_ on age drives higher estimates of the *fpr* in older populations. There was no evidence that BNP levels varied with average patient age among patients with acute heart failure (ratio of means = 1.05, 95% Cr‐I 0.90 to 1.22). Unsurprisingly, given the weak evidence for any association, the model did not lead to any improvement in DIC (Table 2).

***Example 2:* Spot PCR for diagnosis of preeclampsia**

As papers included in this systematic review indicated right skew in values of PCR,^35^, ^36^ we followed the same analysis strategy as for Example 1, ie, first, assuming g = log(). As shown in Table 3, again, the DIC did not provide support for including parameters for between‐study correlations. An extension of the independence model to estimate the most appropriate Box‐Cox transformation parameter (Model 4) provided an estimate of λ = −0.54 (95% Cr‐I ‐0.99, −0.10), indicating that the underlying distributions of test results are slightly more right skewed than log‐logistic. However, this extension to the model was not supported by the DIC, which increased by 4.1 points relative to Model 3.

**Table 3:** Comparison of model fit to the protein to creatinine ratio data, according to the Deviance Information Criteria (DIC). $\bar{D}$= mean residual deviance, pD = effective number of parameters, DIC = $\bar{D}$+ pD

Model	Correlation structure	Transformation, g()	$\bar{D}$	pD	DIC
1	Full correlation matrix	log()	143.5	33.7	177.2
2	Structured correlation matrix	log()	146.1	34.0	180.1
3	Independence	log()	144.9	33.5	178.4
4	Independence	Box‐Cox with unknown *λ*	148.1	34.4	182.5

**Figure 5:**
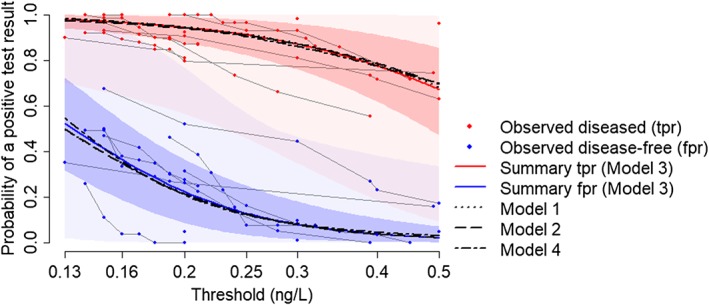
Summary true positive rate (tpr) and false positive rate (fpr) estimates (Models 1‐4) for the protein to creatinine ratio data across the full range of thresholds. 95% credible intervals and prediction intervals shown are from Model 3 [Colour figure can be viewed at http://wileyonlinelibrary.com]

Figure 5 shows that the summary estimates from all four models were very similar across the entire range of thresholds. The maximum absolute difference in *tpr* was 2% (Model 4 versus 3 at the highest thresholds). Summary *fpr* estimates differed by a maximum of 3% across Models 1‐3 but up to 5% for Model 4 versus the others. These discrepancies, as seen on Figure 5, were at the lowest threshold values. In contrast, our summary estimates are markedly different from the best fitting model of Riley et al^15^ (as shown on Figure 2): mean absolute difference in summary *tpr* = 4% (maximum 8%), mean absolute difference in summary *fpr* = 10% (maximum 22%), compared with our Model 3. Our models suggest a much greater dependency of *tpr* and *fpr* on threshold: for example, across the full range of thresholds, summary *fpr*s from the model of Riley et al reduced from 0.30 to 0.02, whereas those from our Model 3 reduced from 0.52 to 0.02. This appears to better capture the range in the observed data.

Prediction intervals are again seen to be extremely wide, reflecting a large amount of between‐study heterogeneity in *tpr* and *fpr* even at the same threshold.

See the supplementay material for a comparison of Model 3 results with results from fitting the model proposed by Steinhauser et al.^4^ Across thresholds, diagmeta consistently estimated a slightly higher summary *tpr* than our Model 3 (median difference = 3%). The Steinhauser model estimated a steeper gradient for the dependence of summary *fpr* on threshold than our model (maximum absolute difference in *fpr* = 9%). 95% CIs around summary estimates from diagmeta were quite different from our 95% Cr‐Is, in particular being narrower at lower thresholds and much wider at higher thresholds for the summary *tpr*. See Discussion for possible explanations.

## 5 DISCUSSION

Since the most appropriate threshold at which to operate a test is usually a key clinical question, there is a need to move beyond “standard” meta‐analysis methods^1^, ^2^, ^3^ to explicitly quantify how sensitivity and specificity vary across thresholds. Perhaps the most obvious approach would be to simply regress logit transformed *tpr*s and *fpr*s (or equivalently, sensitivity and specificity) on *C*. However, we see from Equation (1) that this would imply strong assumptions about the underlying test results: (i) that these have symmetric, logistic distributions; (ii) if assuming constant slope parameters (as is the case in a standard meta‐regression^37^) that the scale parameters of these logistic distributions are constant across studies, which seems unlikely in practice. We have described a model that allows for a range of skewed or symmetric distributions of test results and estimates study‐specific location and scale parameters.

Riley et al^15^ proposed a multivariate normal meta‐regression approach, in which logit (sensitivity) and logit (specificity) are modelled as polynomial functions of threshold. For the PCR data set (our Example 2), they found a cubic relationship with threshold to have the best fit. However, if the common assumption holds that there is a monotonic transformation, g(), that transforms test results in both the diseased and disease‐free populations to logistic, then it follows that logit (sensitivity) and logit (specificity) are in fact linear functions of g(C).

Other approaches have been suggested, which are based on specific assumptions about the underlying distributions of test results.^4^, ^16^ Of these, our proposed model is the most similar to that proposed by Steinhauser et al, who assume that test results have either logistic, log‐logistic, normal, or log‐normal distributions (depending on the choice of link function, logit or probit, and the choice of covariate, *C* or log(*C*)).^4^ Our model allows for a more flexible range of underlying distributions through its ability to estimate a Box‐Cox transformation parameter *λ* within the model. Alternatively, a value of *λ* can be prespecified based on knowledge of the specific test. We note that although *λ* was well estimated in each of our two case studies, computation time was increased (relative to prespecifying *λ* = 0, ie, g() = log()), due to high autocorrelations in simulated values of this parameter.

An implicit assumption of our model is that the same transformation function, g(), applies to all studies in the meta‐analysis. This seems defensible if all studies assessed the same continuous outcome and that this outcome was measured in the same way across all studies. This might not be the case if values are not directly comparable across assays or machines made by different manufacturers. If this is known to be the case, it might be sensible to perform separate analyses for each assay or machine (see Example 1, where we restricted the meta‐analysis to the 18 studies reporting data relating to the same assay).

Our model could also be extended to allow *λ* to vary randomly across studies (similar to the work of O'Malley and Zou^26^) or to estimate a separate transformation parameter for the diseased and disease‐free populations. The latter extension violates the usual assumption made in estimating ROC curves that there is a linear relationship between the *tpr*s and *fpr*s on the logit scale. However, this assumption could be too restrictive, as noted by Putter et al.^14^ For our two case study data sets, neither of these extensions materially impacted on the estimates (not shown).

In addition to increased flexibility in distributional form, our model differs in other ways from that proposed by Steinhauser et al.^4^ It is therefore not surprising that we observed some differences in summary estimates and intervals for both worked examples, even when prespecifying g() = log() (Supplementay material). Firstly, Steinhauser et al made normal approximations to the true multinomial likelihoods of the count data to apply standard linear mixed modelling techniques.^4^ In contrast, we modelled the multinomial likelihoods directly. This automatically accounts for within‐study correlations resulting from a study reporting accuracy measures at more than one threshold and should perform better at thresholds where the number of positive test results is equal to or close to zero.

Likely the primary driver of the differences in these summary estimates, however, is that our between‐study model is quite different from that of Steinhauser et al.^4^ We parameterised our model in terms of the means and scale parameters of transformed test results in the diseased and disease‐free populations. We assumed that the means and log‐transformed scale (log(*σ*_*ij*_)) parameters are normally distributed across studies. In contrast, Steinhauser et al specified logit (*pr*_*i1t*_) and logit (*pr*_*i*2*t*_) as linear functions of *C*_*it*_ or log (*C*_*it*_) and assumed that the intercept and slope parameters were normally distributed across studies.^4^ Note that, by definition, these slope parameters are equal to −1/*σ*_*ij*_ (see Equation (1)). As such, the two models make different assumptions about the nature of the between‐study variation. As the sets of random effects in the different models are not linear transformations of each other, the summary estimates and amount of uncertainty around these may differ (as in the supplementay material). We do not feel that general conclusions on the pattern of differences between the two models can be made based on only two case studies. However, an argument in favour of our parameterisation or between‐studies model is that it automatically constrains all scale parameters, *σ*_*ij*_, to be positive. Steinhauser et al noted in their simulation study that occasionally, their parameterisation leads to estimation of impossible positive slope parameters (equivalently, negative *σ*_*ij*_).^4^

An alternative would be to extend the “hierarchical summary ROC” parameterisation that is often used for meta‐analysis of diagnostic test accuracy.^3^ An extension of this to model multiple thresholds^9^ specifies *pr*_*i*1*t*_ and *pr*_*i*2*t*_ as functions of two sets of random effects (study‐specific “accuracy” and “shape” parameters) and a number of “threshold” parameters, denoted by *θ*_*it*_. The threshold parameters are constrained to be ordered within each study, but are estimated independently of the *θ*_*it*_s in other studies. To extend this model to the case of explicit numerical thresholds, one could instead specify *θ*_*it*_ as a linear function of $g(C_{\text{it}})$, with the intercepts and natural logarithms of the slope parameters being two additional sets of random effects.

The parameterisation in this paper may be the most natural for investigating reasons for between‐study heterogeneity. For example, given that BNP levels have been found to increase with patient age within studies,^33^, ^34^ it was intuitive (in the absence of individual level data) to fit average patient age as a covariate acting directly on the location parameters, *μ*_*ij*_. In other data sets, we might hypothesise that a covariate is more likely to drive differences in the spread of test scores, represented by the scale parameters. Heterogeneity in *fpr* or *tpr* across studies could be driven by differences in either of these sets of parameters. Results of analyses with covariates should be interpreted with the caution advised for any meta‐regression, given likely low statistical power, risk of chance findings and, when modelling the effect of average population characteristics (such as in our worked example), potential ecological bias.^37^

In our two case studies, we found the majority of summary estimates to be reassuringly similar across variations of our model. The BNP analyses illustrate that we should be cautious, however, in interpreting estimates at extreme threshold values with little data. We found no improvement in model fit and very little impact on estimates of target parameters by estimating between‐study correlation parameters relative to estimating each set of random effects separately. This is probably because there is a very little information to inform the between‐study correlations. This will not necessarily be the case in data sets that include large numbers of studies on large numbers of patients. We also note that our “structured” correlation matrix is one of many possible structures that might be hypothesised and fitted, depending on knowledge of the test and data.

Following estimation of “summary” sensitivity and specificity across all thresholds, any one of a number of criteria might be applied to decide upon the optimal threshold. A very simple approach would be to maximise the Youden Index, defined as the sensitivity + specificity – 1.^38^ However, it will not often be appropriate to weight sensitivity and specificity equally in this way. For example, the potential role of natriuretic peptides in an acute care setting is as a “rule out” test: in this context, high sensitivity will generally be considered more important than high specificity.^22^ See Figure 5 of the work of Steinhauser et al^4^ for a demonstration of how weighting the Youden index in favour of sensitivity reduces the “optimal threshold” for BNP testing. One alternative simple approach might be to maximise the sensitivity for a prespecified maximum acceptable *fpr*. More sophisticated approaches explicitly account for the prevalence of the disease in the decision population and the costs and anticipated consequences (good and bad) of all four possible outcomes of a test: true positive, false positive, true negative, false negative. Given an economic decision model, the optimal threshold can be selected to maximise the expected net benefit.^39^

We emphasise the importance of utilising multiple pairs of sensitivity and specificity from studies in a meta‐analysis, where available. Even if these are not stated in the text or tables, it will often be possible to extract additional data from ROC curves using digitized software. These valuable additional data allow for a very flexible modelling approach.

## Supporting information

SIM_8301‐Supp‐0001‐Appendix B.docx

## Data Availability

Data and WinBUGS code for one variation of the model are openly available from https://wiley.figshare.com. WinBUGS code for other variations of the model will be provided by the first author on request.

## PRODUCT NORMAL FORMULATION FOR MULTIVARIATE NORMAL RANDOM EFFECTS

We present a general approach to fitting a multivariate normal distribution of dimension N in Bayesian statistical software using MCMC simulation, such as WinBUGS or OpenBUGS. Consider the following multivariate normal distribution:

$$\left(\begin{array}{l} \theta_{i1}\\ \theta_{i2}\\ \vdots \\ \theta_{\text{iN}} \end{array}\right)∼\text{Normal}\left(\left(\begin{array}{l} \mu_{1}\\ \mu_{2}\\ \vdots \\ \mu_{N} \end{array}\right),\left(\begin{array}{cccc} V_{11} & V_{12} & \text{⋯} & V_{1N}\\ V_{12} & V_{22} & \text{⋯} & V_{2N}\\ \vdots & \vdots & \vdots & \vdots \\ V_{1N} & V_{2N} & \text{⋯} & V_{NN} \end{array}\right)\right).$$

This can be computed more efficiently by rewriting in the equivalent form

$$\left(\begin{array}{l} \theta_{i1}\\ \theta_{i2}\\ \vdots \\ \theta_{\text{iN}} \end{array}\right)=\left(\begin{array}{l} \mu_{1}\\ \mu_{2}\\ \vdots \\ \mu_{N} \end{array}\right)+\left(\begin{array}{l} \delta_{i1}\\ \delta_{i2}\\ \vdots \\ \delta_{\text{iN}} \end{array}\right),$$

where

(A1) $$\left(\begin{array}{l} \delta_{i1}\\ \delta_{i2}\\ \vdots \\ \delta_{\text{iN}} \end{array}\right)∼\text{Normal}\left(\left(\begin{array}{l} 0\\ 0\\ \vdots \\ 0 \end{array}\right),\left(\begin{array}{cccc} V_{11} & V_{12} & \text{⋯} & V_{1N}\\ V_{12} & V_{22} & \text{⋯} & V_{2N}\\ \vdots & \vdots & \vdots & \vdots \\ V_{1N} & V_{2N} & \text{⋯} & V_{NN} \end{array}\right)\right).$$

We could estimate this model by assigning a vague Wishart prior to the precision matrix, but results can be sensitive to the choice of Wishart parameters.^40^ It seems preferable instead to give prior distributions directly to interpretable parameters, such as standard deviation and correlation parameters. This also allows direct comparison of results from our “full correlation matrix” and “structured correlation matrix” models in the main manuscript or any alternative forms of the correlation matrix that might be considered appropriate in a given setting.

However, because the multivariate normal distribution is specified in terms of the precision matrix, it is necessary to invert the covariance matrix at each iteration of the MCMC sampler. Furthermore, at the early stages of the sampler, we may generate covariance matrices that are near singular, and lead to numerical errors when inverted.

We avoid these problems by writing the multivariate normal distribution as a sequence of conditional univariate normal distributions^40^ and deriving the precision matrix iteratively without requiring a numerical inverse procedure.

Define $\Sigma_{n}=\left(\begin{array}{cccc} V_{11} & V_{12} & \text{⋯} & V_{1n}\\ V_{12} & V_{22} & \text{⋯} & V_{2n}\\ \vdots & \vdots & \vdots & \vdots \\ V_{1n} & V_{2n} & \text{⋯} & V_{\mathrm{nn}} \end{array}\right),n=1,\ldots ,N$ such that Σ_1_ = *V*_11_ and the full variance‐covariance matrix is equal to Σ_*N*_. Furthermore, Σ_*n*_ can be partitioned as

$\Sigma_{n}=\left(\begin{array}{cc} \Sigma_{n-1} & v_{.n}\\ v_{.n}^{T} & V_{\mathrm{nn}} \end{array}\right),$ where *v*_.*n*_ = (*V*_1*n*_ *V*_2*n*_ ⋯ *V*_*n* − 1,*n*_)^*T*^, *n* = 2,…,*N*.

Let us further denote $W_{n}=\Sigma_{n}^{-1}$, *n* = 1,…,*N*.

Say that the *δ*_*i*_s are partitioned into two subsets: Δ_*Ai*_ of dimension *p* and Δ_*Bi*_ of dimension *N‐p*. Then, the multivariate normal distribution (A1) can be written as a product of two independent multivariate normal distributions of dimensions *p* and *N‐p*, respectively: the marginal distribution of Δ_*Ai*_, which has mean 0 and covariance matrix simply equal to the relevant partition of Σ_*N*_, and the conditional distribution of Δ_*Bi*_ given Δ_*Ai*_.^41^

Consider the special case where Δ_*Bi*_ = *δ*_*iN*_, ie, *p = N*‐1. Then, (A1) can be written as a product of

$$\left(\begin{array}{l} \delta_{i1}\\ \vdots \\ \delta_{\text{iN}-1} \end{array}\right)∼\text{Normal}\left(\left(\begin{array}{l} 0\\ \vdots \\ 0 \end{array}\right),\Sigma_{N-1}\right)$$

and the following univariate normal distribution for *δ*_*iN*_ conditional on *δ*_(*i*,*N* − 1)_ = (*δ*_*i*1_, …,*δ*_*iN* − 1_)^*T*^:

$$\delta_{\text{iN}}|\delta_{\left(i,N-1\right)}∼\text{Normal}\left(v_{.N}^{T}W_{N-1}\delta_{\left(i,N-1\right)},V_{\text{NN}}-v_{.N}^{T}W_{N-1}v_{.N}\right),$$

ie, $\delta_{\text{iN}}|\delta_{\left(i,N-1\right)}∼\text{Normal}\left(\sum_{i=1}^{N-1}\sum_{j=1}^{N-1}V_{\text{iN}}{\left(W_{N-1}\right)}_{\text{ij}}\delta_{j},V_{\text{NN}}-\sum_{i=1}^{N-1}\sum_{j=1}^{N-1}V_{\text{iN}}V_{\mathrm{jN}}{\left(W_{N-1}\right)}_{\text{ij}}\right).$

As this formula can be applied iteratively, (A1) can be written as a product of conditional univariate normal distributions as follows:

$$\begin{array}{ll} \delta_{1} & ∼\text{Normal}\left(0,V_{11}\right)\\ \delta_{n}|\delta_{1},\ldots ,\delta_{n-1} & ∼\text{Normal}\left(\sum_{i=1}^{n-1}\sum_{j=1}^{n-1}V_{\text{in}}{\left(W_{n-1}\right)}_{\text{ij}}\delta_{j},F_{n-1}\right),\;n=2,\ldots ,N, \end{array}$$

where *F*_*n* − 1_ is the Schur complement of Σ_*n* − 1_,^42^ defined as

$$\begin{array}{ll} F_{n-1} & =V_{\text{nn}}-v_{.n}^{T}W_{n-1}v_{.n}\\ & =V_{\text{nn}}-\sum_{i=1}^{n-1}\sum_{j=1}^{n-1}V_{\text{in}}V_{\text{jn}}{\left(W_{n-1}\right)}_{\text{ij}}. \end{array}$$

Clearly, *W*_1_ = 1/*V*_11_. For *n* = 2,…,*N*, we can find *W*_*n*_ iteratively, as a function of *W*_*n* − 1_, as follows^42^(p80):

$$W_{n}=\left(\begin{array}{cc} W_{n-1}+\frac{1}{F_{n-1}}W_{n-1}v_{.n}v_{.n}^{T}W_{n-1}\; & -\frac{1}{F_{n-1}}W_{n-1}v_{.n}\\ -\frac{1}{F_{n-1}}{\left(W_{n-1}v_{.n}\right)}^{T} & \frac{1}{F_{n-1}} \end{array}\right).$$

Hence, the matrix *W*_*n*_ is defined by the elements:

$$\;{\left(W_{n}\right)}_{\text{nn}}=\frac{1}{F_{n-1}}$$

(A2) $$\;{\left(W_{n}\right)}_{\text{rn}}={\left(W_{n}\right)}_{\text{nr}}=-\frac{1}{F_{n-1}}\sum_{i=1}^{n-1}{\left(W_{n-1}\right)}_{\text{ri}}V_{\text{in}},\;r=1,\ldots ,n-1$$

(A3) $${\left(W_{n}\right)}_{\text{rs}}={\left(W_{n}\right)}_{\text{sr}}={\left(W_{n-1}\right)}_{\text{rs}}+\frac{1}{F_{n-1}}\sum_{i=1}^{n-1}\sum_{j=1}^{n-1}{\left(W_{n-1}\right)}_{\text{is}}{\left(W_{n-1}\right)}_{\text{rj}}V_{\text{in}}V_{\text{jn}},\;r=1,\ldots ,n-1;s=1,\ldots ,n-1.$$

From (A2), we see that (A3) is equivalent to

$${\left(W_{n}\right)}_{\text{rs}}={\left(W_{n}\right)}_{\text{sr}}={\left(W_{n-1}\right)}_{\text{rs}}+F_{n-1}{\left(W_{n}\right)}_{\text{rn}}{\left(W_{n}\right)}_{\text{sn}}.$$
