# Supplementary material for: Quantifying how diagnostic test accuracy depends on threshold in a meta‐analysis
Source: Stat Med. 2019 Sep 30;38(24):4789–803. doi: 10.1002/sim.8301 (PMC6856843; doi:10.1002/sim.8301)
Supplement: Supplementary file 1 — SIM_8301‐Supp‐0001‐Appendix B.docx [file SIM-38-4789-s001.docx]

**Appendix B: Comparison with results from Steinhauser *et al* model**

For each of the two case study data sets in the paper, we compare results from our ‘Model 3’ with results from fitting the model proposed by Steinhauser *et al* (1). The latter was fitted using the R package ‘diagmeta’ ([https://CRAN.R-project.org/package=diagmeta](https://cran.r-project.org/package=diagmeta)). In keeping with our Models 1-3, we assumed that test results in the diseased and disease-free populations had a log-logistic distribution in each study.

In addition to the most general version of their model, ‘DIDS’ (linear mixed effects model with *different random intercepts and different random slopes*), Steinhauser *et al* propose a number of reduced versions (1). We followed Steinhauser *et al* in selecting the variation of the model with the smallest REML criterion. For both data sets, this was the full ‘DIDS’ model.

***Example 1:* B type natriuretic peptide for diagnosis of acute heart failure**


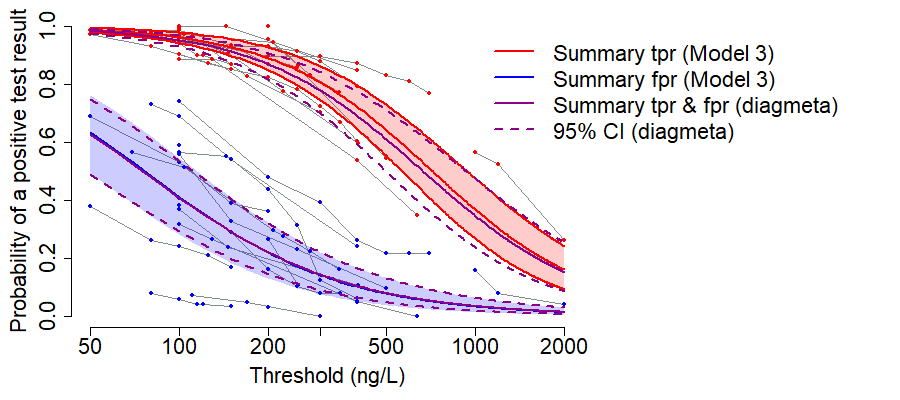


***Figure: Summary true positive rate (tpr) and false positive rate (fpr) estimates from Model 3 (with 95% credible intervals) compared with estimates from the Steinhauser et al ‘DIDS’ model, fitted in diagmeta (with 95% confidence intervals).***

| ***Across all thresholds:*** | ***tpr*** | ***fpr*** |
| --- | --- | --- |
| Median difference in summary estimates:  Model 3 - diagmeta | 0.02 | 0.00 |
| Maximum absolute difference in summary estimates | 0.03 | 0.01 |
| Mean width of 95% credible interval (Model 3) | 0.17 | 0.07 |
| Mean width of 95% confidence interval (diagmeta) | 0.19 | 0.07 |

***Table: Difference in summary tpr and fpr estimates (Model 3 vs Steinhauser et al ‘DIDS’ model, fitted in diagmeta) and comparison of width of 95% credible/confidence intervals***

***Example 2:* Spot PCR for diagnosis of pre-eclampsia**


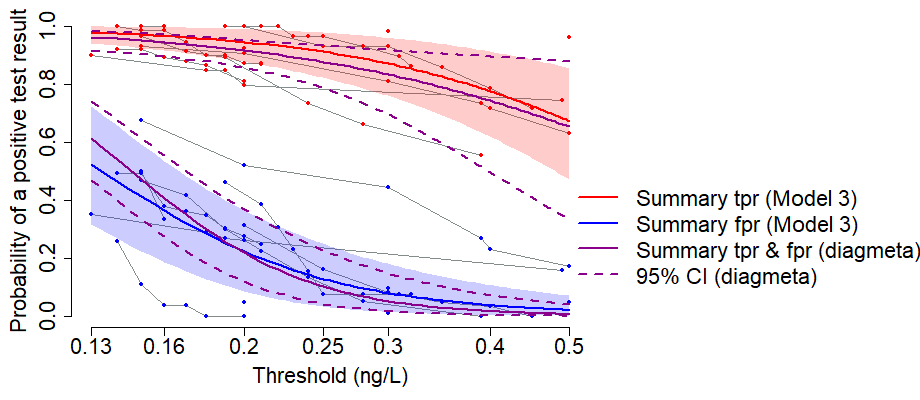


***Figure: Summary true positive rate (tpr) and false positive rate (fpr) estimates from Model 3 (with 95% credible intervals) compared with estimates from the Steinhauser et al ‘DIDS’ model, fitted in diagmeta (with 95% confidence intervals).***

| ***Across all thresholds:*** | ***tpr*** | ***fpr*** |
| --- | --- | --- |
| Median difference in summary estimates:  Model 3 - diagmeta | 0.03 | 0.02 |
| Maximum absolute difference in summary estimates | 0.04 | 0.09 |
| Mean width of 95% credible interval (Model 3) | 0.22 | 0.18 |
| Mean width of 95% confidence interval (diagmeta) | 0.27 | 0.14 |

***Table: Difference in summary tpr and fpr estimates (Model 3 vs Steinhauser et al ‘DIDS’ model, fitted in diagmeta) and comparison of width of 95% credible/confidence intervals***

**References**

1. Steinhauser S, Schumacher M, Rucker G. Modelling multiple thresholds in meta-analysis of diagnostic test accuracy studies. BMC Med Res Methodol. 2016;16(1):97.
